# Identifying new therapeutics for focused ultrasound-enhanced drug delivery in the management of glioblastoma

**DOI:** 10.3389/fonc.2025.1507940

**Published:** 2025-03-13

**Authors:** Ryan Holman, Nathan McDannold

**Affiliations:** Focused Ultrasound Laboratory, Department of Radiology, Brigham and Women’s Hospital, Boston, MA, United States

**Keywords:** drug delivery, focused ultrasound, glioblastoma, glioma, blood-brain barrier, chemotherapy, brain tumors

## Abstract

Glioblastoma, a grade IV astrocytoma, typically has a poor prognosis, with most patients succumbing within eighteen months of diagnosis and few experiencing long-term survival. Focused ultrasound, an emerging localized therapy, has shown promising results in early-phase studies for glioblastoma by improving the uptake of temozolomide and carboplatin. The blood-brain barrier is critical to homeostasis by regulating the movement of substances between the bloodstream and the central nervous system. While this barrier helps prevent infections from bloodborne pathogens, it also hinders the delivery of cancer therapies to gliomas. Combining focused ultrasound with circulating microbubbles enhances local blood-brain barrier permeability, facilitating the intratumoral uptake of systemic cancer therapies. The purpose of this study was to identify promising new therapeutics in the treatment of glioblastoma for localized drug delivery via focused ultrasound. This review provides an overview of the current standard of care for newly diagnosed and recurrent glioblastoma, identifies current therapies indicated for the treatment, discusses key aspects of microbubble resonators, describes focused ultrasound devices under evaluation in human trials, and concludes with a perspective of emerging therapeutics for future studies.

## 1 Introduction

The World Health Organization CNS5 classification categorizes adult glioblastoma multiform (GBM) as isocitrate dehydrogenase wild-type adult-type diffuse astrocytoma with one or more biomarkers, such as necrosis, microvascular proliferation, mutation of the TERT promoter gene, chromosomes +7/-10 copy number changes, or amplification of endothelial growth factor receptor (EGFR) genes (1). Newly diagnosed GBM elicits a poor prognosis with a 14–18 month median overall survival (mOS), a 2-year survival rate of 27%, and a 5-year survival rate of 6% following standard-of-care resection and chemoradiation (2–4). O^6^-methylguanine DNA-methyltransferase (MGMT) methylation presents with a more favorable prognosis, with a 23 month mOS and a 49% 2-year survival rate, relative to a 13 month mOS and 12% 2-year survival rate for MGMT-promoter unmethylated GBM (4).

Focused ultrasound (FUS) holds promise in improving GBM outcomes by enhancing the blood-brain barrier (BBB) permeability to facilitate the localized uptake of systemic therapies. This review explores current and emerging GBM therapies, along with ongoing FUS-enhanced research applications.

## 2 Conventional treatment of newly diagnosed glioblastoma

Newly diagnosed GBM is typically treated with tumor resection preceding concomitant temozolomide (TMZ) chemoradiation and subsequent maintenance (adjuvant) TMZ therapy (4–6). The United States Food and Drug Administration (FDA) has approved five pharmaceuticals and one device for GBM: TMZ, oral lomustine (CCNU), bevacizumab, intravenous carmustine (BCNU), carmustine wafers, and tumor-treating fields (TTFs) (7). Regorafenib and procarbazine hydrochloride-lomustine-vincristine sulfate (PCV) combination therapy are also listed in the United States National Comprehensive Cancer Network (NCCN) guidelines as preferred treatments for recurrent GBM (rGBM) (2, 8).

Resection is often limited to tumor debulking and histological sampling (4). GBM tumors can grow along vessels and fiber tracts microscopically several centimeters beyond the macroscopic tumor region (9, 10). Early glioma hemispherectomies saw recurrence in the contralateral hemisphere (11). Radiotherapy targets the excision cavity and remnant tumor sites, typically with 2 Gy fractions totaling 60 Gy over 6 weeks, concurrently with TMZ (2, 12).

TMZ is an alkylating agent activated at physiological alkalinity to 5-(3-methyl)-1-triazen-1-yl-imidazole-4-carboxamide (MTIC) within approximately 2 hr of oral administration, and passively diffuses across vascular endothelial cell membranes (13–17). TMZ levels in brain parenchyma are typically less than 20% of blood plasma (18–21). Improved outcomes occur with MGMT-promoter methylated GBM, where epigenetic silencing by methylation of CpG (5’—Cytosine—phosphate—Guanine—3’) sites within the MGMT gene promotor region reduces the reparation of TMZ-induced alkylation (22, 23).

TTFs with maintenance TMZ were FDA-approved and incorporated into NCCN guidelines after improving median progression-free survival (mPFS) and mOS for newly diagnosed GBM (NCT00916409) (2, 24, 25). The Optune Gio (Novocure, Haifa, Israel) is FDA-approved for recurrent and newly diagnosed GBM (26). Alternating electric fields of 0.7 V.cm^-1^ and 200 kHz create dielectrophoretic movement of charged organelles and dipolar macromolecules to induce cell death of proliferating tumor cells (27–30).

## 3 Treatment for recurrence

**Figure 1:**
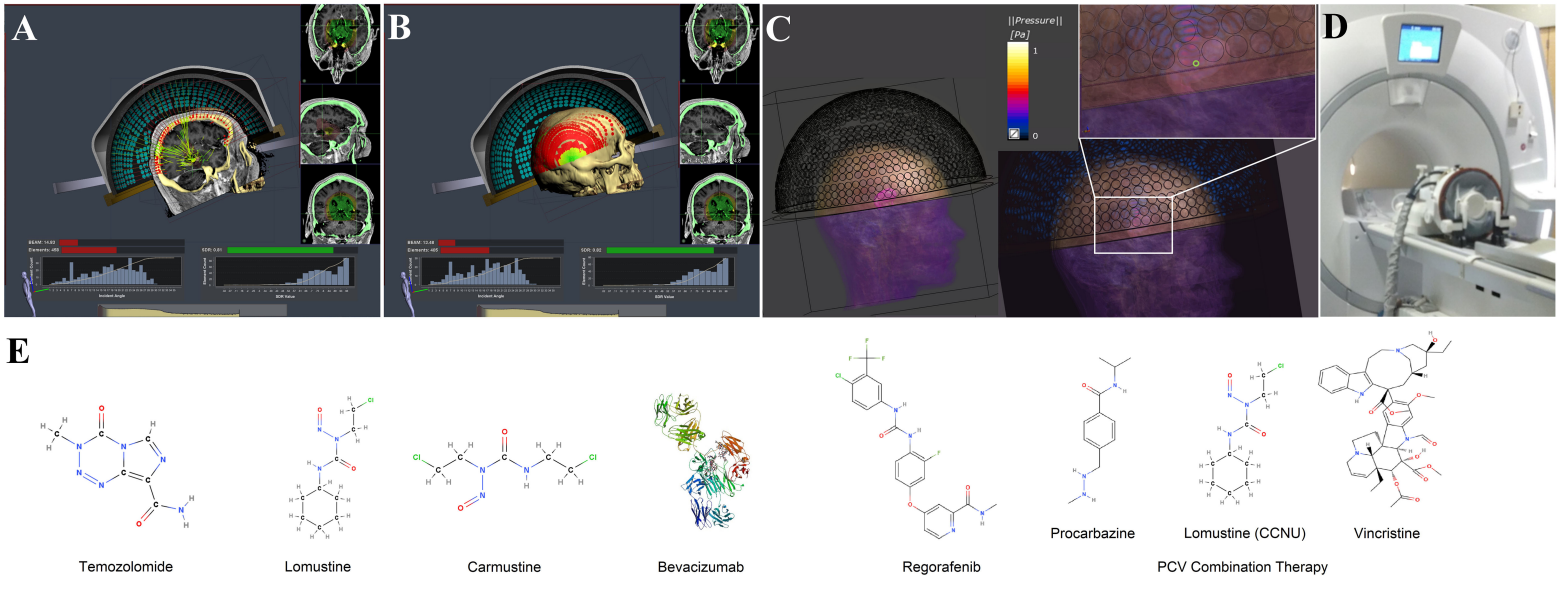
This illustration provides an overview of a hemispherical array used in blood-brain barrier (BBB) opening procedures, along with the molecular structures of selected glioblastoma therapies. **(A)** An example of a peripheral tumor site, which leads to high incidence angles for many elements during ray tracing. The application is intended for ablative treatments to deactivate elements, but illustrates the extent of incidence angles beyond 30°. **(B)** An illustration of the incidence angle distribution in relation to the skull surface. The red circles represent elements with incidence angles more than 30°, and the green circles represent those less than 30°. Figure made with Kranion and datasets from The Cancer Imaging Archive (182, 183). **(C)** A simulated normalized pressure field for a 220 kHz Exablate 4000 Type 2.0 transducer model, without aberration correction, recreated using settings described previously (184). **(D)** An image of an Exablate 4000 Type 1.0 transducer system used for ablative procedures. **(E)** The molecular structure of pharmaceuticals approved by the United States Food and Drug Administration (FDA) or recommended by the United States National Comprehensive Cancer Network (NCCN) for the treatment of glioblastoma (185, 186).

Recurrence rates and mOS are about 90% and 7–9 months, respectively (12, 31, 32). rGBM treatment options include further surgical resection, TMZ rechallenge, alkylating agents, PCV chemotherapy, re-irradiation, bevacizumab, TTFs, regorafenib, palliative care alone, and experimental techniques (2, 12). Molecular structures and pharmacological properties of GBM therapeutics are shown in **Figure 1E** and **Table 1**.

**Table 1:** Pharmacological Information of Selected Therapeutics to Treat Glioblastoma Multiforme.

Metric	Temozolomide (13, 15, 43, 187, 188)	Bevacizumab (38, 43, 189–192)	Lomustine (CCNU) (36, 43, 193–196)	Carmustine (BCNU) (43, 196–200)	Regorafenib (34, 201)
Active Metabolites	MTIC	unknown	cis-4-hydroxy-CCNU trans-4-hydroxy-CCNU	2-chloroethyl isocyanate	M-2 M-5
Excretion Route	feces 1% urine 38%	unknown	urine 50%	respiration 10% urine 65%	feces 71% urine 19%
Dose	concomitant 75 mg.m^-2^ for 42 d adjuvant 150 mg.m^-2^ daily for 5 d of a 28 d cycle	10 mg.kg^-1^ every 2 wk	130 mg.m^-2^ every 6 wk	150-200 mg.m^-2^ every 6 wk	160 mg daily for 3 wk of a 4 wk cycle
C_max_	temozolomide 7.5 µg.mL^-1^ (38.6 µM) MTIC 282 ng.mL^-1^ (1.5 µM)	284 µg.mL^-1^ (1.9 µM)	cis-4-hydroxy-CCNU 0.3 µg.mL^-1^ (1.2 µM) trans-4-hydroxy-CCNU 0.5 µg.mL^-1^ (2.0 µM)	1.0 µg.mL^-1^ (4.7 µM) (530 mg.m^-2^)	2.5 µg.mL^-1^ (5.0 µM)
t_1/2_	1.8 hr	20 d	cis-4-hydroxy-CCNU 1.3-2.9 hr trans-4-hydroxy-CCNU 1.3-2.5 hr	22 min (530 mg.m^-2^)	M-2 25 hr M-5 51 hr regorafenib 28 h
AUC	temozolomide 23.4 µg.hr.mL^-1^ MTIC 0.9 µg.hr.mL^-1^	3.2 mg.d.mL^-1^	cis-4-hydroxy-CCNU 0.8–1.6 µg.hr.mL^-1^ trans-4-hydroxy-CCNU 1.4–2.3 µg.hr.mL^-1^	4.1 µg.hr.mL^-1^ (530 mg.m^-2^)	70.4 µg.hr.mL^-1^
Lipinski Rule of 5	Yes	No	Yes	Yes	Yes
Molecular Weight	194.2 Da	149 kDa	233.7 Da	214.1 Da	500.8 Da
GI_50_	100 µM	NA	31.6 µM	52.1 µM	8.0 µM
LC_50_	U87 82.3 µM U87-MGMT >200.0 µM	NA	328 µM	173 µM	unknown

AUC, mean plasma area under the curve; CCNU, chloroethyl-cyclohexyl-nitrosourea; C_max_, maximal blood plasma levels; GI_50_, concentration needed for 50% cell growth inhibition; LC_50_, concentration needed for 50% cell death; M-2, regorafenib N-oxide; M-5, N-desmethyl-regorafenib; MTIC, 5-(3-methyl)1-triazen-1-yl-imidazole-4-carboxamide; t_1/2_, mean elimination half-life; TMZ, temozolomide.

Regorafenib is an anti-angiogenic multi-kinase (VEGFR1-3, TIE2) inhibitor added to NCCN rGBM guidelines after improving mOS compared to lomustine (2, 33, 34). TTFs are a chemotherapy-free treatment option that can improve toxicity and quality of life (2, 35). Lomustine is the *de facto* standard, with improved outcomes for MGMT-promoter methylated GBM, and is frequently a control arm in clinical trials, with a 2 months mPFS, 20% 6-month PFS, and a mOS of 6–9 months (36). Anti-angiogenic bevacizumab is a monoclonal antibody that neutralizes circulating vascular endothelial growth factors (VEGF) (2, 37, 38). Debate exists regarding improved mOS, but the treatment can reduce steroid use and enhance quality of life (39).

## 4 The blood-tumor barrier

The BBB provides an interface between the brain parenchyma and capillaries, regulating homeostasis by managing blood flow, oxygenation, glucose, essential amino acids, and other metabolite levels (40, 41). During progression, the BBB changes can be distinguished as the blood-tumor barrier (BTB), which features disrupted tight and adherens junctions, extensive BBB fenestration, and inhibition of receptor-mediated transcellular pathways (40, 42). Natural BTB disruption enhances BBB permeability, but drugs often remain less than ten times higher than in healthy brain tissue (40). The BTB allows the passage of small ions and molecules but restricts the entry of larger therapeutics (18, 40, 41). Lipinski’s rule of 5 predicts passive BBB permeability, indicating that no more than one of the following criteria can be violated: less than six hydrogen bond donors, less than eleven hydrogen bond acceptors, a molecular weight of less than 500 Da, and a lipophilicity octanol-water partition coefficient less than five (43, 44).

## 5 Focused ultrasound-mediated blood-brain barrier opening

### 5.1 Microbubbles

Microbubbles are used off-label as resonators for BBB opening. Optison (GE Healthcare, Chicago, IL, USA), SonoVue/Lumason (Bracco S.P.A., Milan, Italy), and Definity/Luminity (Lantheus Medical Imaging, North Billerica, MA, USA) have received FDA approval for contrast-enhanced ultrasonography (45, 46). Human FUS studies have often used Definity and SonoVue microbubbles (47).

Clinical FUS studies with the Exablate Neuro have been performed with both microbubble bolus doses (48) and infusion rates of 0.24–0.8 µL.kg^-1^.min^-1^ (49, 50), with possible treatment durations around 3 hr (50). Mechanical index (MI) thresholds for stable and inertial cavitation are temperature and tissue-dependent (51). The *in vivo* thresholds with Definity for FUS-enhanced BBB opening and inertial cavitation have been measured near 0.46 and 0.72–1.15, respectively (52, 53). The mean diameters for Definity microbubbles are 1.1–3.3 µm, with 98% less than 10 µm, and 100% less than 20 µm (54). Definity bolus doses exhibit a mean blood plasma half-life of 1.3–1.9 min, achieve intravascular equilibrium within 1 min, have a maximum serum concentration near 2 min, and become undetectable after 10–14 min (54–56). The C_3_F_8_ gas is inert, with low solubility, is eliminated non-metabolized through the lungs, and, in the presence of dissolved respiratory gas allows extended dissolution rates (57). The shell reduces perfluorocarbon gas diffusion, prevents coalescence, and reduces the immune response (57).

The microbubble mechanisms of BBB opening are believed to be independent of bulk heating and inertial cavitation (58). The acoustic radiation force propels the microbubbles toward the capillary walls, where microbubble oscillations trigger events, including shear stresses and microstreaming, that culminate in BBB opening (41, 58, 59). The enhanced drug uptake occurs largely through disturbance of the tight junctions, dysregulation of efflux transporters, and increased caveolae formation (60). BBB closure occurs over approximately 4–6 hr, with complete closure within 24 hr (58, 61). Influential factors include the MI, microbubble dose, duty cycle, vessel to bubble diameter ratio, frequency, tissue properties, and sonication duration (58, 62). Many cavitation-related bioeffects remain unknown, such as effects from microjetting, reactive oxygen species, ballistic motion, and bubble clusters (62).

Functionalizing microbubbles and altering their shape and size can prolong the circulatory half-life and improve drug delivery to smaller capillaries for more uniform concentrations (63). Nanobubbles increase disruption in smaller 2–6 µm rodent capillaries (64). Antibody-microbubble conjugates can target microscopic metastatic brain tumor sites for use with large-volume ultrasound fields (65). Perfluorocarbon droplets are similar colloids to microbubbles with a liquid rather than gaseous core and have shown potential for drug delivery (66). Droplets are size-tunable (67), integrate chemotherapeutics (68), prolong systemic circulation (69), increase inertial cavitation thresholds (70), enable ^19^F MRI (71), and potentially exhibit a unique cavitation mechanism (72). Other formulas incorporate metal chelates (73, 74), allow partial oxygen measurements in gliomas (75–77), can track macrophages after re-irradiation in glioma-bearing mice (78), and incorporate within clinical cell therapies for cell tracking (79, 80) and measuring apoptotic cell fraction (81). Nanodroplets have also exhibited the ability to permeabilize the BBB (82–84).

### 5.2 FUS devices

Devices and drugs for BBB disruption include FUS, laser ablation, mannitol, RMP-7, and regadenoson (85). Other localized drug delivery approaches include convection-enhanced delivery, intra-arterial catheter delivery, reservoir implants, stereotactic injections, and carmustine wafers placed in the resection cavity (86).

Thermoablative procedures are the only FDA-approved FUS modalities for neurological disease, and the 670 kHz Exablate Neuro 4000 Type 1.0/1.1 (InSightec, Haifa, Israel) is the only system both FDA-approved and Conformité Européenne (CE)-marked (87). Additional research applications include hyperthermia, sonothrombolysis, neuromodulation, histotripsy, sonodynamic therapy, and liquid biopsy (60, 87). At least three FUS devices have been used in early-phase clinical trials for BBB opening in GBM, including the NaviFUS (NaviFUS Corp., Taipei, Taiwan), Exablate Neuro 4000 Type 2.0 (InSightec, Haifa, Israel), and SonoCloud-9 implant (CarThera, Paris, Île-de-France, France) (47, 60, 88–90).

The 220 kHz Exablate Neuro 4000 Type 2.0 system is a hemispherical phased array transducer with ±25 mm electronic steering and treatment volumes beyond 30 cm^2^ (49, 91). Ray tracing aberration correction incorporates the shear sound speed with incidence angles beyond 30° (91). The transducer integrates with existing neuroablation systems and can treat conditions beyond GBM. Repeated BBB opening during maintenance TMZ has illustrated prolonged survival, with no adverse events or TMZ neurotoxicity (NCT03712293) (19, 48). Elevated concentrations have been observed with liposomal doxorubicin, TMZ, and fluorescein (NCT02343991, NCT03322813) (92, 93). The device has completed Phase 2 trials for sonodynamic therapy in newly diagnosed GBM (NCT04845919) and is ongoing for carboplatin monotherapy for rGBM (NCT04417088, NCT04440358). A safety and feasibility study was recently completed for maintenance TMZ in newly diagnosed GBM (NCT03551249) (49, 91, 94).

Hemispherical arrays are monitored with embedded acoustic receivers for microbubble harmonic emissions. Numerous approaches have been developed for feedback control (95). The Exablate algorithm is proprietary but generates a cavitation score from the harmonic emissions, and allows altered sonication duration, applied power, gain, and cavitation dose goal (49). Human GBM studies observed lower microbubble concentrations than in animals, the need for improved receiver sensitivity, and relatively hypovascular white matter targets that reduced microbubble concentrations (49). Sites near the skull surface can lead to standing waves, reflections, and impact focusing (49). **Figures 1A–D** illustrates the system and the incidence angle distributions at a peripheral target site.

The Sonocloud-9 is a 1 MHz MRI-compatible, minimally invasive, transcranial implant placed in the location of the bone flap after tumor resection or biopsy (96, 97). Clinical studies include carboplatin for rGBM (NCT03744026), checkpoint inhibitors for metastases (NCT04021420), carboplatin for pediatric gliomas (NCT05293197), nanoparticle albumin–bound paclitaxel (nab-paclitaxel) for rGBM (NCT04528680), anti-programmed cell death protein 1 (aPD-1) and anti-cytotoxic T-lymphocyte-associated protein 4 (aCTLA-4) monoclonal antibodies and liposomal doxorubicin in newly diagnosed GBM (NCT05864534), and adjuvant TMZ for newly diagnosed GBM (NCT04614493) (96, 98–100). The device can target large volumes (∼45 cm^2^), features short procedure times, and is not influenced by skull aberration (89). Thus avoiding aberration correction, MRI guidance, feedback control, and can be performed on an outpatient basis (88, 101–103). Safety and feasibility studies showed tolerability and evidence of improved mOS for carboplatin delivery in rGBM (NCT02253212, NCT03744026) (96, 98, 103–105), and a Phase 3 trial is underway (NCT05902169).

NaviFUS is a 500 kHz 256-channel neuronavigational phased array system attached to a mechanical arm designed to be used without a stereotactic headframe (NCT03626896, NCT04446416, NCT04988750) (106–109). Studies have used ramped-up feedback control at 0.5–0.68 MI (107, 109). Position sensors register the device to pretreatment imaging for 3D focal tracking. The system integrates intraoperative pressure simulations, lowers cost, increases portability, with treatment durations below 15 min, and negates intraoperative MRI guidance (88, 108, 109). The device has evaluated enhanced bevacizumab delivery for rGBM (NCT04446416) (109) and illustrated a possible synergistic effect with radiotherapy (NCT04988750) (107). An rGBM Phase 3 trial is evaluating bevacizumab delivery (NCT06496971).

Previously suggested technical improvements include whole-brain electronic steering, cavitation mapping, simulation-based focusing, and holography (91, 110). Passive acoustic mapping has been limited by axial resolution (109) but could be correlated with bioeffects, tumor response, and local drug concentrations (62, 111). The approach is feasible with neuronavigational systems (112, 113) and hemispherical arrays (114, 115). Receiver arrays within custom hemispherical transducers could enable MRI-free procedures (116, 117). Diagnostic extra-cranial systems have been adapted for acoustic mapping for drug delivery to colorectal liver metastases (ISRCTN17598292) (118).

### 5.3 Pharmaceuticals in development with focused ultrasound

Thorough lists of GBM clinical trials and preclinical studies evaluating a range of therapeutics are provided elsewhere (47, 88–90, 119–122). Briefly, therapeutics evaluated in animal models include TMZ, methotrexate, irinotecan, carboplatin, paclitaxel, carmustine, doxorubicin, cisplatin, etoposide, MGMT inactivators, targeted therapies like bevacizumab, and immunotherapies like checkpoint inhibitors and CAR T-cell therapy. Many of these drugs are used off-label and have been evaluated by systemic administration or loading within nanocarriers and microbubbles (119). Pharmaceuticals evaluated in clinical studies include TMZ, doxorubicin, liposomal doxorubicin, aPD-1 antibodies, aCTLA-4 antibodies, fluorescein, bevacizumab, paclitaxel, nab-paclitaxel, and carboplatin (90, 96, 99, 100, 120). FUS-mediated BBB opening is also being evaluated for Parkinson’s disease (50, 123, 124), Alzheimer’s disease (125–132), amyotrophic lateral sclerosis (133), and metastatic brain tumors (134).

FUS can modulate the innate immune response, improve the penetrance of targeted therapies and immunotherapies, and improve survival in rodents (99, 100, 119, 122). A number of immunotherapies are being evaluated clinically in combination with FUS for primary and secondary brain tumors. Balstilimab, botensilimab, and pembrolizumab are being studied for newly diagnosed and rGBM (NCT05864534) (99, 100). Pembrolizumab is being assessed in a Phase 3 trial for non-small cell lung cancer brain metastases (NCT05317858). Nivolumab, pembrolizumab, and ipilimumab are being evaluated for melanoma brain metastases (NCT04021420).

Drug-loaded microbubble and nanocarriers, along with drug conjugates, offer alternatives to systemic administration (111, 119). Nanoparticle therapeutics increase preclinical survival times and relative concentrations than systemic antibodies and chemotherapies (111). The nanocarrier hydrodynamic diameters ideally remain below 100 nm (119, 135), with a trade-off between increased permeation and clearance rates (136). Nanocarrier ligand groups target vascular or tumor surface receptors and allow internalized and externally activated drug release (119).

### 5.4 Challenges to translation

Overcoming the BBB is the main challenge to GBM therapies (137). Difficulties in focused ultrasound adoption include establishing standardized treatment settings and rigorous safety studies (138). Hypovascular white matter targets reduce drug delivery (49, 105). Further, concurrent anesthetic administration can alter hemodynamics, vasoactivity, and temperature to confound permeability and cavitation thresholds (63). New GBM animal models are needed to better account for surgical resection, recurrence, and immunological response (119). The disease is rare, with about 8–11% clinical trial participation (7), and only three pivotal studies between 2005–2022 prolonged survival (139, 140). With hemispherical arrays, individual patient characteristics can influence outcomes, such as skull characteristics on feedback control (49). Ablation is complicated by bone attenuation, impedance mismatch, skull heating, and bone heterogeneity (141, 142). These aspects are less problematic for BBB opening because the lower frequencies and powers reduce acoustic absorption, aberrations, and risk of thermal damage (142, 143).

## 6 Emerging GBM therapeutics

At least two additional therapeutic regimens have improved mOS in Phase 3 trials in recent years, but had contentious trial designs (144, 145). Autologous tumor lysate-dendritic cell vaccine (DCVax-L) reported improved mOS for newly diagnosed and recurrent GBM (NCT00045968) (146, 147). Lomustine-TMZ combination therapy improved mOS for newly diagnosed MGMT-promoter methylated GBM compared to standard-of-care chemoradiation (NCT01149109) (148). TTFs with maintenance TMZ arguably provide the best survival rates in newly diagnosed GBM (24), and a pragmatic approach would be evaluating FUS-enhanced adjuvant TMZ with TTFs (119). TTFs with Withaferin A illustrated a synergistic effect, suggesting increased vulnerability to anti-mitotic chemotherapies (30, 149, 150). Immunotherapies have mostly lacked survival benefits in Phase 3 trials (151). New targets such as immunosuppressive CD73 myeloid cells have been proposed with anti-CD73 antibodies in combination with aCTLA-4 and aPD-1 therapies (152–154).

Theranostics can measure longitudinal pharmacokinetics, biodistribution, and drug concentrations for association with treatment response (155). R_1_ relaxation rates and volumetric transfer coefficients are surrogates for drug concentration (156–158). *In vivo* radiolabeled GBM therapeutics can be quantitatively imaged with nuclear imaging, with or without FUS, using ^11^C-TMZ (half-life: 20.3 min) (159), ^68^Ga-bevacizumab (half-life: 68 min) (160, 161), and ^89^Zr-cetuximab (half-life: 78.4 hr) (162, 163). ^89^Zr-bevacizumab has been evaluated without FUS in pediatric diffuse intrinsic pontine glioma (164), a condition under evaluation for FUS-mediated drug delivery (165). Radionuclide therapeutics for metastatic prostate cancer and somatostatin receptor-positive gastroenteropancreatic neuroendocrine tumors received regulatory approval with [^177^Lu]Lu-PSMA-617 and [^177^Lu]Lu-DOTA-TATE, respectively (166, 167). These therapies along with [^131^I]-IPA, [^177^Lu]Lu-NeoB, [^177^Lu]Lu-FF58, and [^177^Lu]Lu-6A10-Fab fragments are in clinical trials for GBM (166, 168, 169). Carrier-mediated L-type amino acid transporters (LAT-1) such as small molecule [^131^I]-IPA (NCT03849105, NCT05450744) have high BBB permeability and would allow quantitative comparison of drug delivery with FUS (137, 170–173). [^14^C]-regorafenib has been used in human pharmacokinetic studies (174), and 3.0 T and 9.4 T ^19^F-MRI has illustrated longitudinal measurements of trifluoro-methylated pharmaceuticals, similar to regorafenib, in murine models (175, 176). Fluorine-containing metastatic chemotherapies with less than two rule of 5 violations include abemaciclib (177), larotrectinib, encorafenib, and vemurafenib (178). Larotrectinib has no rule of 5 violations (178), has shown promise for pediatric neurotrophic tyrosine receptor kinase (NTRK) fusion-positive gliomas (179, 180), and in adults NTRK gene fusions are most frequently found in GBM (181).

## 7 Discussion

While most studies have been to establish safety and feasibility, limiting inclusion criteria to MGMT-promoter methylated GBM for FUS-enhanced TMZ therapies could improve outcomes due to epigenetic silencing. TTFs, lomustine-TMZ combination therapy, and DCVax-L have demonstrated improved mOS in Phase 3 trials of newly diagnosed GBM, and have not been evaluated in conjunction with FUS. Studies of FUS-enhanced drug delivery with lomustine have been relatively limited. ^19^F-MRI might allow for longitudinal drug concentrations in preclinical survival studies, using regorafenib, larotrectinib, drug-loaded perfluorocarbon nanodroplets, or cell therapies labeled with perfluorocarbon emulsions. Radionuclide theranostics like LAT-1 [^131^I]-IPA or [^177^Lu]Lu-DOTA-TATE could be used similarly with nuclear imaging.

In conclusion, FUS-enhanced delivery of systemic therapies has demonstrated safety, tolerability, and evidence of efficacy in preclinical and early-phase clinical studies and presents a promising localized delivery technique with the potential to improve the standard-of-care management for GBM.
